# The Evaluation of Oral Health Condition and Oral and Dental Care in Children with Congenital Heart Disease

**DOI:** 10.3390/jcm12113674

**Published:** 2023-05-25

**Authors:** Fatma Saraç, Sera Şimşek Derelioğlu, Fatih Şengül, Fuat Laloğlu, Naci Ceviz

**Affiliations:** 1 Department of Pedodontics, Faculty of Dentistry, Atatürk University, 25240 Erzurum, Türkiye; simseksera@gmail.com (S.Ş.D.); fatihs@gmail.com (F.Ş.); 2 Department of Pediatrics, Division of Pediatric Cardiology, Faculty of Medicine, Atatürk University, 25240 Erzurum, Türkiye; fuatlaloglu@atauni.edu.tr (F.L.); ceviznaci@yahoo.com.tr (N.C.)

**Keywords:** congenital heart disease, oral health, enamel defects, hypoxia

## Abstract

**Objective:** Congenital heart disease (CHD) plays a key role in oral and dental health regarding its own impacts on teeth (i.e., enamel hypoplasia), infective endocarditis and choice of dental treatment. The purpose of this study’s comparing the oral and dental health status in children with or without CHD is to contribute to the literature by determining the effects of CHD on oral and dental health. **Material and Methods:** The present study was conducted using a descriptive and correlational design and consisted of 581 children aged between 6 months and 18 years who were healthy (n = 364) or experienced CHD (n = 217). CHD-impacted children were classified according to their shunt and stenosis and then their saturation values were noted. In the intraoral examination, caries data (dmft/DMFT, PUFA/pufa), oral hygiene (OHI-S) and enamel defect (DDE) indices were recorded. Statistical analyses were performed using SPSS 26.0 at a significance level of 0.05. **Results:** In our study, caries index scores of children with or without CHD in primary or permanent dentition were found to be similar. The mean OHI-S index (*p* < 0.001) and gingivitis findings (*p* = 0.047) of children with CHD had a higher prevalence than the healthy ones. The incidence of enamel defects was determined as 16.5% in CHD-affected children whereas an incidence rate of 4.7% was observed in healthy children. The mean saturation value of the participants with enamel defects (89 ± 8.9) was observed to be significantly lower (*p* = 0.03) than the patients with no enamel defects (95 ± 4.2). **Conclusions:** Whereas the caries index scores of CHD-affected children with a history of hypoxia in primary and permanent dentition were found to be similar to the healthy ones, children with CHD were observed to be more prone to enamel defects and periodontal diseases. Furthermore, considering the risk of infective endocarditis resulting from existing carious lesions and periodontal problems, it is highly important for pediatric cardiologists, pediatricians and pediatric dentists to collaborate in a multidisciplinary manner.

## 1. Introduction

Congenital heart disease (CHD) is a structural abnormality of the heart or intrathoracic great vessels during intrauterine development. It occurs in approximately 0.8–1% of all live births. Patients with CHD may be acyanotic or cyanotic. In the presence of CHD, development of many organs, tissues and systems can be adversely affected depending on the severity of the pathology. One of these structures is dental tissues [1,2,3]. Oral health and risk factors are often neglected as parents focus on chronic diseases [1]. It has been reported that children with CHD had more carious lesions [1,2], more dental plaque and gingivitis [4] and therefore had worse oral health than healthy children. Identifying and eliminating dental problems in children with CHD or preventing the emergence of these problems with preventive health services is very important in increasing patients’ quality of life.

Tooth formation and timing of amelogenesis may overlap in children with CHD. This leads to hypoplasia and hypomineralization due to the reduction in enamel deposition in the teeth [1]. Reviewing the literature, it is observed that children with CHD have a higher rate of enamel defects than healthy children [5,6]. However, the number of studies on the etiology of this condition is limited.

Correlation between oral health and dental health has been widely accepted in recent years and the number of corroborating studies has also been on the rise. Furthermore, it has been revealed that periodontal treatments had a positive effect on glycemic control and cardiovascular diseases [7,8]. Dental caries and periodontal diseases may lead to or aggravate systemic disorders [9]. Cariogenic microorganisms enter the bloodstream through dental and supporting tissues and may trigger inflammatory reactions. These microorganisms may also induce IE, which has already been frequently seen in CHD patients [10,11,12]. Higher prevalence of enamel defects observed in children with CKD than healthy ones may result in caries whereas higher gingivitis incidence may increase susceptibility to periodontal diseases as well intensify the risk of IE. Thus, it is important to perform routine dental checkups and necessary treatments and implement oral hygiene in children experiencing CHD.

The aim of the current study was to assess the oral and dental health status of children experiencing various types of CHD who were treated and followed-up in the department of pediatric cardiology and also to compare them with healthy ones. Our null hypothesis was established as “Oral and dental health conditions of children with different types of CHD are not different from those of healthy ones”.

## 2. Materials and Methods

This study was a descriptive and correlational cross-sectional study. Our study was carried out between November 2021 and April 2022 and approval was obtained from the Ethics Committee of Ataturk University Faculty of Medicine (B.30.2.ATA.0.01.00/39). This study was conducted in accordance with the Declaration of Helsinki and informed consent forms were obtained from all parents.

### 2.1. Patient Selection

This study consisted of one study group including 217 CHD-impacted children (112 boys and 105 girls) and one control group involving 364 healthy children (177 boys and 187 girls). Study group comprised children who were diagnosed with CHD and followed-up in Atatürk University Faculty of Medicine, Department of Pediatric Cardiology whereas the control group included the healthy children from the pre, primary, middle and high schools affiliated with Erzurum National Education Directorate.

#### 2.1.1. Inclusion Criteria

Willing patients between 6 months and 18 years old with a CHD diagnosis who were followed-up and/or their parents were included in the study group.

Children of the same age group with no systemic disorders and/or their parents accepting to join were involved in the control group.

#### 2.1.2. Exclusion Criteria

Any systemic diseases and disorders affecting bone metabolism, thyroid and adrenal hormone disorders or cirrhosis; patients with chronic renal failure (glomerular filtration rate (eGFR) < 30 mL/min/1.73); patients receiving corticosteroid therapy, vitamin D, parathormone and calcium metabolism disorders; patients with gonadal pathology; patients receiving heparin therapy and antiepileptics, with a history of drugs affecting bone tissue or current drug use; and patients using chemotherapeutic agents were not included in our study.

#### 2.1.3. Sample Size Calculation

In the main hypotheses of this research, we planned to investigate the differences and relationships between the independent groups, and the sample size was calculated at a 95% confidence level using the G Power 3.1.9.2 program [13]. According to the analysis result, with a standardized effect size of 0.1839 obtained from the previous study [3] (GI 82% and 64.8% at α = 0.05 and with a theoretical power of 0.95), the minimum sample size was calculated as 385.

### 2.2. Data Collection

#### 2.2.1. Clinical Cardiological Evaluation

The current CHD guideline is accepted as a reference in the diagnosis, classification (Table 1) and treatment of children with CHD [14]. A professor in pediatric cardiology with a 25 years of experience reviewed the medical history of the patients. The history of surgery or transcatheter cardiovascular intervention and hemodynamic significance of the pathology at the date of the oral examination were noted.

Oxygen saturations of the patients were measured using a fingertip saturation device (NellcorTM Bedside SpO2 Patient Monitoring System, Medtronic, Minneapolis, MN, USA). Medications used by the patients, if any, were recorded. SpO2 ≤ 92% in room air was recorded as significant regarding the low oxygen saturation. Demographic data and clinical information were obtained during routine examinations.

The criteria for hemodynamic significance in patients with CHD with left-to-right shunt were as follows: a shunt ratio (Qp/Qs) > 2 for ventricular septal defect, >1.5 for atrial septal defect and a typical murmur for patent ductus arteriosus or presence of symptoms indicating that the left ventricle was under volume load in the echocardiographic evaluation. All cyanotic congenital heart diseases were considered hemodynamically significant. Presence of criteria requiring treatment in CHD with stenosis was considered to be hemodynamically important [14,15].

#### 2.2.2. Clinical Oral Health Assessment

Dental examinations of the patients were performed using a flat mouth mirror and a blunt-tipped probe with the help of optimal compressed air and lighting. Decayed, missing and filling indexes of primary teeth (dmft) and permanent teeth (DMFT) were calculated according to the World Health Organization (WHO) criteria [16]. The results of pulpal involvement due to carious teeth were evaluated using PUFA/pufa indexes [17]. Each child’s oral hygiene was measured using the simplified oral hygiene index (OHI-S). According to the OHI-S measurement values, 0.0–0.6 was graded as good oral hygiene, 0.7–1.8 as moderate and 1.9–3.0 as poor [18]. In the evaluation of developmental enamel defects, the DDE index was preferred. The codes used in this index were as follows: 0 (without defect), 1 (demarcated opacity), 2 (diffuse opacity), 3 (hypoplasia), 4 (other defects), 5 (combination of diffuse and demarcated opacities), 6 (combination of demarcated opacities and hypoplasia), 7 (combination of diffuse opacities and hypoplasia), 8 (combination of all defects), and 9 (excluded) [19].

### 2.3. Statistical Analysis

In this study, descriptive statistics (percentage, mean, frequency and standard deviation) were calculated as the first step of data analysis. The suitability of the data to the normal distribution was investigated using the Kolmogorov–Smirnov test and graphing methods. In the analysis of the numerical variables conforming to the normal distribution, the independent sample t-test was used to compare two independent groups while the one-way analysis of variance (one-way ANOVA) was used to compare three or more independent groups. The post-hoc Tukey test was performed in multiple comparisons. For non-normally distributed numerical variables, the Mann–Whitney U test was used to compare two independent groups and the Kruskal–Wallis test was used to compare three or more independent groups. Relationships between categorical variables were examined using the Pearson chi-square test when the sample size assumption was met (n > 5). Relationships between numerical and ordinal variables were checked using Kendall’s tau correlation. Statistical analysis was performed using SPSS software version 26.0 (SPSS Inc., Chicago, IL, USA) at a significance level of 0.05.

## 3. Results

A total of 217 children (112 boys and 105 girls) were included in the study group whereas the control group involved a total of 364 children (177 boys and 187 girls) with a similar intergroup gender distribution (*p* = 0.49). The mean age of both groups, 8.9 ± 4.8 and 9.27 ± 3.71 years old, respectively, was not statistically different (*p* = 0.325). Of the patients in the study group, 45 had cyanotic and 172 had acyanotic CHD.

The mean dmft/dft/DMFT and dmfs/dfs/DMFS indexes, PUFA/pufa indexes and OHI-S scores of the groups are shown in Table 2. In the mixed dentition period, the mean DMFS score in the study group (5.7 ± 5) was significantly higher than the control group (4 ± 3.4) (*p* = 0.031). Again, in the mixed dentition period, the mean dfs score of the control group (10.7 ± 7.4) was statistically significantly higher than that of the study group (8.2 ± 5.8) (*p* = 0.047). In the primary dentition, the mean pufa score (3 ± 1.4) of the control group was significantly higher than that of the study group (1.5 ± 0.5) (*p* = 0.039).

The frequency of enamel defects detected in the groups is shown in Table 3. The frequency of enamel defects in the study group was significantly higher than in the control group (*p* < 0.001).

The frequency of enamel defects in CHD subgroups is shown in Table 4. A statistically significant difference was observed between healthy children and CHD subgroups with RL shunt, and in subgroups with stenosis by the frequency of enamel defects (*p* < 0.05).

In terms of enamel defect types, the average number of teeth with enamel defects in the disease groups is shown in Table 5. Since DDE index codes 7 and 8 were not observed in any patient, they were not included in the table. The mean number of teeth with limited opacity was significantly higher in the subgroups of hemodynamically significant CHD with LR shunt, increased/decreased pulmonary blood flow CHD with RL shunt and hemodynamically unimportant CHD with stenosis than in the healthy group (*p* < 0.001).

The mean number of teeth with diffuse opacity in the CHD subgroup with reduced pulmonary blood flow with RL shunt was significantly higher than in all groups except the CHD subgroup with increased pulmonary blood flow with RL shunt and the hemodynamically significant CHD subgroup with stenosis. In addition, the mean number of teeth with diffuse opacity was significantly higher in the hemodynamically significant CHD subgroups with stenosis and in CHD subgroups with reduced pulmonary blood flow with an RL shunt than in the healthy group (*p* < 0.001).

Regarding the mean number of teeth affected, the CHD group with decreased pulmonary blood flow with RL shunt differed significantly from healthy individuals and hemodynamically significant and nonsignificant CHD subgroups with LR shunt. Regarding the mean number of affected teeth, the hemodynamically significant CHD subgroup with LR shunt was similar to the healthy individuals but significantly lower than all other disease subgroups (*p* < 0.001).

The mean saturation values of children in the study group were found to be significantly lower in those with enamel defects (89 ± 8.9) and those without enamel defects (95 ± 4.2) (*p* = 0.03). There was no statistically significant difference in the presence of enamel defects between the patients with acyanotic and cyanotic CHD in the study group (*p* = 0.121). In addition, no statistically significant relationship was observed between the hemodynamically significant CHD and the presence of enamel defect (*p* = 0.843).

## 4. Discussion

Many studies have examined the oral and dental health of children with CHD. The effects of CHD on oral–dental health were grouped under three main headings in the studies: the effects of the disease on the teeth, the risk of infective endocarditis (IE) and the indication for treatment when dental intervention is required [20]. Our study aimed to evaluate a gap in the literature by evaluating the oral hygiene status of children with CHD and to examine the relationship between enamel defects and disease groups.

Information on the prevalence of caries in children with CHD differs. Studies report that children with CHD have a higher, similar, statistically insignificant but a lower prevalence of caries than healthy children [2,3,21,22]. In our study, during the mixed dentition period, there was a statistically significant difference between higher DMFS score of the study group and higher dfs score in the control group. Tooth extraction, a more radical procedure, is preferred for deep caries lesions with pulpal involvement in primary teeth of the CHD-impacted patients with a risk of IE [12]. For this reason, in our study, the mean dfs scores in which the missing primary teeth in the mixed dentition were excluded may have been lower in the group with CHD. Although all other mean caries index scores in the mixed dentition were found to be higher in children with CHD, they did not show statistical significance. Higher caries prevalence was reported in a recent study conducted in Erzurum [23,24]. In our study, carried out in the same region, higher caries prevalence on par with that of children with no systemic diseases observed in CHD-impacted children was also found to be risky for developing IE.

When children with CHD and healthy ones were compared, no statistically significant difference was found between PUFA/pufa indices in the primary, mixed and permanent dentition. Regarding IE, similar PUFA/pufa indices, which should be much lower in children with CHD in all dentition periods, is extremely risky for healthy children.

In most cases, parents of children experiencing CHD do not assign priority to oral health, rather focusing on chronic diseases [25], which in turn might result in similar PUFA/pufa index scores observed in both CHD-impacted and healthy children in our study. However, higher PUFA/pufa scores may also pose life-threatening risks for children with CHD.

Different results have been reported regarding the plaque index (PI), another parameter evaluated in our study. Studies have revealed that children with CHD have a higher or similar PI than healthy children [2,3,26,27,28]. In our study, mean PI values were found to be significantly higher in children with CHD in all dentition periods (*p* < 0.001). The literature review demonstrated that there were different opinions about the effect of CHD on gingivitis [29,30]. Stelman et al. [31] showed that oral colonization of specific HACEK microorganisms was higher in children with CHD than healthy children and gingivitis was more common in children with CHD. In our study, the frequency of gingivitis was found to be higher in children with CHD than in healthy ones (*p* = 0.047).

A study investigating parental awareness of their children’s oral health and dietary habits revealed that the parents of CHD-impacted children had an unacceptable level of ignorance regarding the importance of oral health [25]. In a study that assessed the oral health of children with CHD in western Norway, Sivertsen et al. [2] remarked that 1/3 of the children suffering from CHD had oral conditions that might pose a risk for systemic disorders. Similarly, in a study conducted in Türkiye, evaluating the knowledge and awareness of the CHD-impacted children’s parents on the relationship between oral and dental health and IE, Yilmaz et al. revealed that 35.4% of the parents did not recognize this correlation [32]. Although not assessed in our study, higher mean values of dental plaque and presence of gingivitis might have been associated with poor parental awareness of the IE and CHD relationship.

Studies have shown that children with CHD develop atherosclerosis earlier and have a higher risk of developing vascular diseases and complications when they reach adulthood [33,34]. This increased risk among children with CHD is also associated with increased systemic inflammatory mediators following chronic gingivitis [35].

Hallet et al. reported that the prevalence of enamel defects was higher in children with CHD (52%) than the control group [27]. In a study by Al Etbi et al. [5], comparing the presence of enamel defects in the primary teeth of patients with ventricular septal defect (VSD) and the healthy group, they found that enamel defects were significantly higher in the VSD group. In our study, DDEs were found to be statistically higher in children with CHD than in the healthy group (*p* < 0.001).

To the best of our knowledge, there are no comprehensive studies on the etiology of enamel defects in children with CHD in the literature. During oxidative stress, cells respond with hypoxia-inducible factor 1 (HIF-1), which mediates repair and adaptation mechanisms. An in vitro study illustrated that, under hypoxic conditions, HIF expression increased in ameloblasts [36]. In another in vitro study, in which tooth germs were incubated for one week under hypoxic conditions, delayed amelogenesis was observed and after four weeks the mineral density decreased by approximately 20% [37]. Dentin mineralization is controlled by various molecular events such as dentin sialophosphoprotein and dentin matrix protein-1 (DSPP, DMP1). DMP1 and DSPP expressions were decreased in dentin and odontoblasts in hypoxic conditions compared to normoxic conditions [38]. Significant differences in ameloblast reaction were observed due to exposure of the ameloblasts to severe hypoxia in mouse incisors, which in turn resulted in hypoplasia and hypomineralization defects in the enamel [36]. For this reason, the relationship between the saturation values and enamel defects in children with CHD was investigated in the present study. In our study, the mean saturation value of children with CHD was found to be statistically significantly lower in those with enamel defects (89 ± 8.9) than in those without enamel defects (95 ± 4.2) (*p* = 0.03). In addition, the prevalence of enamel defects in children with CHD showed a different distribution according to disease types. The mean number of teeth with enamel defects in the control group was similar to the hemodynamically significant CHD subgroup with LR Shunt (acyanotic CHD) whereas it was significantly lower than in all other CHD disease subgroups. Furthermore, the mean number of teeth with enamel defects in the CHD group with decreased pulmonary blood flow with an RL shunt (cyanotic CHD) was higher than in that of healthy children and children with other CHD types. These may be associated with the lower saturation value of CHD with an RL shunt and CHD with stenosis than in healthy children [39]. These findings support a relationship between enamel defects and hypoxia, which is rated as one of etiological factors for DDE but not yet entirely proven.

In our study, higher OHI-S scores and gingivitis rates were found in children suffering from CHD, although similar caries index values were found for both primary and permanent dentition. Oral and dental health conditions of children with different forms of CHD were observed to be different from healthy ones. Thus, our null hypothesis of “oral and dental health conditions of children with different types of CHD are not different from those of healthy ones” was rejected. Although this study reveals the importance of oral and dental health for children with CHD, further long-term multicenter studies with a large number of late-operated or nonoperated patients are needed to clearly reveal the correlation between hypoxia and enamel hypoplasia.

Assessing a substantial number of our patients with hemodynamically significant CHD following cardiac corrective procedures might have a negative effect on our results. Moreover, since recent developments in the field of pediatric cardiology have allowed for treatments before tooth eruption (approximately 6 months of age), it is difficult to find patients with ongoing hemodynamic burden. Because patients with cyanotic CHD can now be operated on early in life, hypoxic exposure time is reduced. Since CHD is a rare disease, the relatively small patient portfolio might also have adversely affected our results.

## 5. Conclusions

The importance of performing dental examinations in children with CHD, especially following eruption in pediatric dentistry clinics, has been demonstrated once again. Additionally, considering the risk of IE due to existing caries and periodontal problems, it is recommended that pediatric cardiologists, pediatricians and pediatric dentists work in a multidisciplinary manner.

## Figures and Tables

**Table 1:** Classification of congenital heart diseases.

**CHDs with Left-to-Right (LR) Shunts**
Not hemodynamically significant
Hemodynamically significant
**CHDs with Right-to-Left (RL) Shunts**
Diseases in which pulmonary blood flow is increased
Diseases in which pulmonary blood flow is reduced
**CHD with Stenosis (with or without Valve Regurgitation)**
Hemodynamically significant
Not hemodynamically significant

**Table 2:** The distribution of caries indices according to the dentition periods of children (mean ± standard deviation).

		Study Group	Control Group	*p*
**Primary Dentition**	**dmft**	5.4 ± 4	5 ± 3.4	0.583
	**dmfs**	9 ± 8.5	9.7 ± 9	0.693
	**pufa**	1.5 ± 0.5	3 ± 1.4	0.039
	**OHI-S**	0.7 ± 0.7	0.03 ± 0.1	<0.001
**Mixed Dentition**	**dft**	5.1 ± 3.2	4.7 ± 2.9	0.532
	**dfs**	8.2 ± 5.8	10.7 ± 7.4	0.047
	**DMFT**	3.1 ± 1.7	2.6 ± 1.7	0.161
	**DMFS**	5.7 ± 5	4 ± 3.4	0.031
	**pufa**	1.4 ± 0.6	2 ± 1.4	0.238
	**PUFA**	1.7 ± 0.9	1.6 ± 0.5	0.837
	**OHI-S**	1.3 ± 0.7	0.08 ± 0.2	<0.001
**Permanent Dentition**	**DMFT**	4.1 ± 2.5	4 ± 2.7	0.841
	**DMFS**	7.9 ± 6.1	6.2 ± 5.5	0.151
	**PUFA**	1.5 ± 0.9	1.4 ± 0.5	0.698
	**OHI-S**	1.3 ± 0.7	0.06 ± 0.2	<0.001

dmft = decayed–missing–filled teeth index for primary teeth; DMFT = decayed–missing–filled teeth index for permanent teeth; OHI-S = simplified oral hygiene index; dmfs = decayed–missing–filled surface score (primary teeth); DMFS = decayed–missing–filled surface score (permanent teeth); PUFA/pufa = pulpal involvement (P/*p*), ulceration (U/u), fistula (F/f), abscess (A/a).

**Table 3:** Distribution of children in study and control groups by the presence of enamel defect (n (%)).

Enamel Defect	Study Group	Control Group	Total
**Yes**	36 (16.5)	17 (4.7)	53 (9.1)
**No**	181 (83.5)	347 (95.3)	529 (90.9)
**Total**	217 (100)	364 (100)	581 (100)

**Table 4:** Distribution of healthy children and CHD subgroups by the presence of enamel defect (n (%)).

Enamel Defect	Healthy	Hemodynamically Insignificant CHD with LR Shunt	Hemodynamically Significant CHD with LR Shunt	CHD with Increased Pulmonary Blood Flow with RL Shunt	CHD with Decreased Pulmonary Blood Flow with RL Shunt	Hemodynamically Significant CHD with Stenosis	Hemodynamically Insignificant CHD with Stenosis	Total
**Yes**	17 ^b^ (5)	6 ^a,b^ (14)	6 ^a,b^ (11)	4 ^a^ (22)	7 ^a^ (25)	7 ^a^ (22)	6 ^a^ (19)	53 (9)
**No**	347 ^b^ (95)	36 ^a,b^ (86)	60 ^a,b^ (90)	14 ^a^ (78)	21 ^a^ (75)	25 ^a^ (78)	25 ^a^ (81)	527 (91)
**Total**	364 (100)	42 (100)	66 (100)	18 (100)	28 (100)	32 (100)	31 (100)	581 (100)

The difference between groups marked with different letters on the same line is significant (*p* < 0.05).

**Table 5:** Distribution of the mean number of teeth with enamel defect in the disease groups according to the enamel defect types of children (mean ± standard deviation).

	Healthy	Hemodynamically Unimportant CHD with LR Shunt	Hemodynamically Significant CHD with LR Shunt	CHD with Increased Pulmonary Blood Flow with RL Shunt	CHD with Decreased Pulmonary Blood Flow with RL Shunt	Hemodynamically Significant CHD with Stenosis	Hemodynamically Insignificant CHD with Stenosis	*p*
**Limited opacity**	0.1 ± 0.4 ^a^	0.4 ± 1.1 ^a,b^	0.5 ± 2.6 ^b^	1 ± 2.1 ^b^	0.7 ± 1.9 ^b^	0.4 ± 1.2 ^a,b^	0.6 ± 2.1 ^b^	<0.001
**Diffuse opacity**	0 ± 0.1 ^bc^	0.1 ± 0.5 ^b^	0.3 ± 1.3 ^b^	0.4 ± 1.5 ^a,b^	1.3 ± 4.2 ^a^	0.7 ± 2.5 ^a,d^	0.2 ± 1.1 ^b^	<0.001
**Hypoplasia**	0 ± 0.3	0.1 ± 0.6	0.1 ± 0.5	0 ± 0	0.1 ± 0.8	0.1 ± 0.6	0.1 ± 0.7	0.298
**Combinations**	0 ± 0	0.1 ± 0.5	0.1 ± 0.7	0 ± 0	0.1 ± 0.4	0.1 ± 0.2	0 ± 0	0.145
**Demarcated and diffuse**	0 ± 0	0 ± 0	0.1 ± 0.7	0 ± 0	0 ± 0	0 ± 0	0 ± 0	0.263
**Demarcated and hypoplasia**	0 ± 0 ^a^	0.1 ± 0.5 ^b^	0 ± 0 ^a^	0 ± 0 ^a^	0.1 ± 0.4 ^b^	0.1 ± 0.2 ^b^	0 ± 0 ^a^	0.003
**Number of teeth affected**	0.1 ± 0.5 ^a^	0.5 ± 1.6 ^a,c^	0.8 ± 3.4 ^c^	1.4 ± 2.8 ^b,c^	2 ± 5.2 ^b^	1.1 ± 3.1 ^b,c^	1 ± 2.9 ^b,c^	<0.001

The difference between groups marked with different letters on the same line is significant (*p* < 0.05).

## Data Availability

The data supporting the findings of this study are available from the corresponding author upon reasonable request.
